# Genetic diversity and antimicrobial resistance profiles of *Staphylococcus pseudintermedius* associated with skin and soft-tissue infections in companion animals in Lisbon, Portugal

**DOI:** 10.3389/fmicb.2023.1167834

**Published:** 2023-04-17

**Authors:** Catarina Morais, Sofia Santos Costa, Marta Leal, Bárbara Ramos, Mariana Andrade, Carolina Ferreira, Patrícia Abrantes, Constança Pomba, Isabel Couto

**Affiliations:** ^1^Global Health and Tropical Medicine, GHTM, Instituto de Higiene e Medicina Tropical, IHMT, Universidade NOVA de Lisboa, UNL, Lisbon, Portugal; ^2^Laboratory of Antibiotic Resistance, CIISA, Faculty of Veterinary Medicine, University of Lisbon, Lisbon, Portugal; ^3^GeneVet, Laboratório de Diagnóstico Molecular Veterinário, Carnaxide, Portugal

**Keywords:** *Staphylococcus pseudintermedius*, SSTIs, antimicrobial resistance, clonal lineage, MLST, companion animals, pets

## Abstract

*Staphylococcus pseudintermedius* is the main bacterial pathogen of skin and soft-tissue infections (SSTIs) in companion animals. Antimicrobial resistance in this species is a growing public health concern. This study aims to characterize a collection of *S. pseudintermedius* causing SSTIs in companion animals, establishing the main clonal lineages and antimicrobial resistance traits. The collection corresponded to all *S. pseudintermedius* (*n* = 155) causing SSTIs in companion animals (dogs, cats and one rabbit) collected between 2014 and 2018 at two laboratories in Lisbon, Portugal. Susceptibility patterns were established by disk diffusion for 28 antimicrobials (15 classes). For antimicrobials without clinical breakpoints available, a cut-off value (CO_WT_) was estimated, based on the distribution of the zones of inhibition. The *blaZ* and *mecA* genes were screened for the entire collection. Other resistance genes (e.g., *erm*, *tet*, *aadD, vga*(C)*, dfrA*(S1)) were searched only for those isolates showing an intermediate/resistance phenotype. For fluoroquinolone resistance, we determined the chromosomal mutations in the target genes *grlA* and *gyrA*. All the isolates were typed by PFGE following *Sma*I macrorestriction and isolates representative of each PFGE type were further typed by MLST. Forty-eight out of the 155  *S. pseudintermedius* isolates (31.0%) were methicillin-resistant (*mecA*^+^, MRSP). Multidrug-resistant (MDR) phenotypes were detected for 95.8% of the MRSP and 22.4% of the methicillin-susceptible (MSSP) isolates. Of particular concern, only 19 isolates (12.3%) were susceptible to all antimicrobials tested. In total, 43 different antimicrobial resistance profiles were detected, mostly associated with the carriage of *blaZ*, *mecA*, *erm*(B), *aph3-IIIa*, *aacA-aphD, cat*_pC221_, *tet*(M) and *dfr*(G) genes. The 155 isolates were distributed within 129 PFGE clusters, grouped by MLST in 42 clonal lineages, 25 of which correspond to new sequence types (STs). While ST71 remains the most frequent *S. pseudintermedius* lineage, other lineages that have been replacing ST71 in other countries were detected, including ST258, described for the first time in Portugal. This study revealed a high frequency of MRSP and MDR profiles among *S. pseudintermedius* associated with SSTIs in companion animals in our setting. Additionally, several clonal lineages with different resistance profiles were described, evidencing the importance of a correct diagnosis and selection of the therapy.

## 1. Introduction

*Staphylococcus pseudintermedius* is a coagulase-positive staphylococci described in 2005 by Devriese et al. (2005), which belongs to the *Staphylococcus intermedius* group (SIG) together with *Staphylococcus intermedius*, *Staphylococcus delphini* and *Staphylococcus cornubiensis* (Bannoehr and Guardabassi, 2012; Murray et al., 2018). *S. pseudintermedius* is the main bacterial pathogen responsible for skin and soft tissue infections (SSTIs) in companion animals (Loeffler and Lloyd, 2018). These infections are often associated with superficial or deep layers of the skin or in the follicular hair (pyoderma).

*S. pseudintermedius* is one of the main bacterial colonizers of the canine skin and mucous membranes. It is an opportunistic pathogen, present in 46–92% of healthy dogs, and responsible for up to 92% of canine pyoderma cases (Lynch and Helbig, 2021). In cats, this bacterium is not part of the normal flora of the skin, but the few available data indicate that in healthy felines, the frequency of *S. pseudintermedius* detection may vary from 2.5 to 8.8% (Ma et al., 2020; Bierowiec et al., 2021) and in sick cats from 2.1 to 12.5% (Qekwana et al., 2017; Saputra et al., 2017; Bierowiec et al., 2019).

Methicillin-resistant *S. pseudintermedius* (MRSP) are resistant to all beta-lactam antimicrobials, except to 5th generation cephalosporins like ceftaroline (Schwarz et al., 2018; Clinical and Laboratory Standards Institute (CLSI), 2022). This is due to the acquisition of the Staphylococcal Chromosomal Cassette *mec* (SCC*mec*), which carries the *mecA* gene (Katayama et al., 2000) responsible for beta-lactam resistance. This cassette may also transport other resistance genes. This and other mobile genetic elements may lead to multidrug resistance patterns (MDR). MRSP strains are also frequently resistant to aminoglycosides, macrolides, lincosamides, fluoroquinolones, trimethoprim-sulfamethoxazole, tetracyclines and chloramphenicol (Pires dos Santos et al., 2016). In Europe, the available data indicate an MRSP frequency of 12.2% in Germany (Feßler et al., 2022), 14.0% in Finland (Grönthal et al., 2017), 16.9% in France (Haenni et al., 2014) and 31.6% in Italy (Menandro et al., 2019). In Portugal, the first MRSP was described in 2007 and since then has been increasingly detected (Couto et al., 2016). In 2011, Couto and colleagues reported 6.2% MRSP isolates among *S. pseudintermedius* isolated in Lisbon (Couto et al., 2011) and in 2016, a study with *S. pseudintermedius* associated with pyoderma, otitis and urinary tract infections from companion animals collected in the same region between 1999 and 2014 revealed a frequency of 8.7% MRSP (Couto et al., 2016). Recently, another study performed in the North of Portugal between 2013 and 2021 showed a ~ 6% frequency rate of MRSP associated with skin infections in companion animals (Garcês et al., 2022). Furthermore, the study conducted by Couto et al. (2016) revealed an increasing trend of antimicrobial resistance between 1999 and 2014 to mostly all main antimicrobials classes and *mecA* carriage in staphylococci causing infections in companion animals.

Several *S. pseudintermedius* clones have been described around the world. ST71 is the most disseminated and commonly found in Europe (Bergot et al., 2018). This lineage comprises exclusively MRSP-MDR isolates. However, in the last decade, lineage ST258, which belongs to clonal complex CC258 has emerged in Europe (Haenni et al., 2014; Damborg et al., 2016; Duim et al., 2016; Grönthal et al., 2017; Ventrella et al., 2017; Swedres-Svarm, 2020; Ruiz-Ripa et al., 2021) and the data suggest that it is gradually replacing ST71 in some of these countries (Duim et al., 2016). This lineage usually includes MRSP or methicillin-susceptible *S. pseudintermedius* (MSSP) isolates with a lower burden of antimicrobial resistance (Pires dos Santos et al., 2016; Bergot et al., 2018; Rynhoud et al., 2021). Other relevant lineages include ST68, prevalent in North America, and ST45 and ST112, prevalent in Asia (Pires dos Santos et al., 2016). In Portugal (Lisbon), the most frequent lineage until 2014 was ST71 associated with MRSP-MDR isolates (Couto et al., 2016). Recently, Silva and colleagues suggested the clonal replacement of ST71 by ST123, a single locus variant (SLV) of ST71 (Silva et al., 2021).

Pyoderma is the primary cause for antimicrobial prescription and the most frequent skin infection detected in companion animals (Loeffler and Lloyd, 2018; Lynch and Helbig, 2021). The recommended treatment for canine pyoderma relies on topical antimicrobials, biocides and, when necessary, systemic antimicrobials (Beco et al., 2013; Hillier et al., 2014; Morris et al., 2017). The simultaneous and inappropriate use of these substances in human and animal practice leads to the emergence of resistant strains. Pyoderma treatment is a challenge in veterinary medicine due to increasing antimicrobial resistance and limited antimicrobials approved for use in companion animals. These issues have led to increasing prescription of critically or highly important antibiotics used in human and veterinary medicine (World Organisation for Animal Health (OIE), 2019; World Health Organisation (WHO), 2019; European Medicines Agency, 2020).

In addition, staphylococci may share their genetic information within and across species and genera and there is a growing concern regarding *S. pseudintermedius* as a reservoir for antimicrobial resistance genes (Wendlant et al., 2013), and is already recognized as a public health hazard (Pomba et al., 2017).

Considering the scarce knowledge on *S. pseudintermedius* epidemiology in Portugal and the increasing conditioning factors for pyoderma treatment, this study aimed to characterize a collection of *S. pseudintermedius* causing SSTIs in companion animals collected over 5 years in Portugal’s capital, Lisbon, with the main goal to detail the genetic diversity and antimicrobial resistance traits associated with this species.

## 2. Materials and methods

### 2.1. Bacterial isolates

The study collection comprised 155 *S. pseudintermedius* isolated from SSTIs in 141 dogs, three cats and one rabbit (Supplementary Table 1). These isolates corresponded to all the SSTI-related *S. pseudintermedius* isolated and identified between 2014 and 2018 at a research laboratory from a veterinary teaching hospital (Lab 1, n = 90) and during 2018 at a private veterinary diagnostic clinic (Lab 2, *n* = 65), both in Lisbon, Portugal. *S. pseudintermedius* isolated from the same animal were only included if pheno- and/or genotypically distinguishable; these corresponded to 18 isolates from the same animals as follows: 12 isolates collected from six dogs (two isolates/dog) plus six isolates collected from two dogs (three isolates/dog).

Bacteria were grown in Tryptone-Soya Agar (Oxoid Ltd., Basingstoke, United Kingdom) or Tryptone-Soya Broth (Oxoid Ltd) at 37°C and total DNA was extracted by the boiling method (Alexopoulou et al., 2006). Briefly, isolated bacterial colonies were suspended in TSB and grown overnight at 37°C. Then, 1 ml of the bacterial culture was centrifuged and the pellet was resuspended in 10 mM Tris, 1 mM EDTA (TE) buffer, 1% (v/v) Triton X (Sigma-Aldrich, St Louis, MO, United States) and proteinase K (0.18 mg/l, Sigma-Aldrich) for the cell lysis (1 h at 56°C). After boiling, the suspension was centrifuged and DNA collected from the supernatant and kept at −20°C for further analysis. Species identification was confirmed by PCR amplification of a *S. pseudintermedius*-specific fragment (198 bp) of the *spsJ* gene (Verstappen et al., 2017), using as a positive control total DNA of strain *S. pseudintermedius* DSM21284^T^ and as negative controls total DNA from strains *S. aureus* ATCC^®^29213™, *S. epidermidis* ATCC^®^12228™ and *S. coagulans* DSM6628^T^. The primers used are described in Supplementary Table 2. Each reaction contained NZYTaq II buffer (1x), NZYTaq II DNA polymerase (0.75 U) (NZYTech, Lisbon, Portugal), dNTPs NZYMix (0.2 mM) (NZYTech), MgCl_2_ (1.75 mM), 0.4 μM of each primer and template DNA. All PCR were performed in a Biometra Uno II (Analytik Jena, Jena, Germany) or Eppendorf™ Mastercycler Personal 5332 (Eppendorf, Hamburg, Germany). All PCR products were analyzed by 2% (w/v) agarose gel electrophoresis, stained with GreenSafe Premium (NZYTech) and visualized under UV light in a Gel-Doc XR (Bio-Rad, Hercules, CA, U.S.A.) apparatus.

### 2.2. Antimicrobial susceptibility testing

The susceptibility of each isolate was assayed against a panel of 28 antimicrobials from 15 antimicrobial classes by the Kirby-Bauer method in Mueller-Hinton agar (MHA, Oxoid Ltd.) using the following disks (Oxoid Ltd., or MAST Group Ltd., Bootle, UK): beta-lactams [penicillin (10 U), oxacillin (1 μg), cefoxitin (30 μg)], tetracyclines [tetracycline (30 μg), minocycline (30 μg), tigecycline (15 μg)], macrolides [erythromycin (15 μg)], lincosamides [clindamycin (2 μg)], streptogramins [quinupristin-dalfopristin (15 μg)], aminoglycosides [gentamycin (10 μg), kanamycin (30 μg), amikacin (30 μg), neomycin (30 μg), tobramycin (10 μg), apramycin (15 μg)], fluoroquinolones [enrofloxacin (5 μg), pradofloxacin (5 μg), ciprofloxacin (5 μg), moxifloxacin (5 μg)], phenicols [chloramphenicol (30 μg), florfenicol (30 μg)], oxazolidinones [linezolid (30 μg)], trimethoprim-sulfamethoxazole (25 μg), fusidic acid (10 μg), rifampicin (5 μg), bacitracin (10 U), novobiocin (30 μg), and mupirocin (200 μg). The D-zone test was performed to detect inducible clindamycin resistance. Presumptive beta-lactamase production was inferred by observation of the zone of inhibition border for penicillin disks. Susceptibility profiles were interpreted according to Clinical and Laboratory Standards Institute (VET01S CLSI) (2020) (clindamycin, enrofloxacin, pradofloxacin and tetracycline), M100-ED32 (2022) (penicillin, oxacillin, gentamicin, erythromycin, minocycline, ciprofloxacin, moxifloxacin, trimethoprim-sulfamethoxazole, chloramphenicol, rifampicin, and linezolid) or European Committee on Antimicrobial Susceptibility Testing (EUCAST) (2023) (quinupristin-dalfopristin, tigecycline and fusidic acid). For isolates showing a discrepancy between oxacillin resistance and *mecA* carriage (*n* = 3), oxacillin minimum inhibitory concentration (MIC) was determined using MIC Test Strip (Liofilchem, Roseto degli Abbruzzi, Italy) in MHA plates and the corresponding susceptibility profiles evaluated according to CLSI guidelines. For further analysis, isolates categorized as intermediate by CLSI were considered together with resistant isolates. Isolates showing resistance to at least one antimicrobial of at least three classes of antimicrobials were considered multidrug resistant (Sweeney et al., 2018). The control strains used for disk diffusion were *S. aureus* ATCC^®^25923™ and *S. aureus* ATCC^®^29213™. The antimicrobials tested were selected according to their relevance for SSTIs therapy or the epidemiology of antimicrobial resistance in *S. pseudintermedius*.

For the antimicrobials with no available breakpoints (amikacin, apramycin, kanamycin, neomycin, tobramycin, novobiocin, florfenicol, mupirocin and bacitracin), a cut-off value (CO_WT_) was estimated based on the distribution of the zones of inhibition through the Normalized Resistance Interpretation (NRI) method (Kronvall et al., 2003; Kronvall and Smith, 2016). For each species-antimicrobial combination, the NRI method uses the distribution of the zones of inhibition to perform a least-square regression analysis and calculate the wild-type (WT) population, the mean zone of inhibition, the associated standard deviation (SD) and the CO_WT_ (2.5X the SD above the mean value and rounded up to the lowest absolute value; Kronvall et al., 2003; Kronvall and Smith, 2016). The CO_WT_ corresponds to the lowest zone of inhibition presented by the WT population, only validated for distributions with an SD < 3.38 mm. The NRI method was used with permission from the patent holder, Bioscand AB, TÄBY, Sweden (European patent No 1383913, US Patent No. 7,465,559). The automatic and manual excel programs were made available through courtesy by P. Smith, W. Finnegan and G. Kronvall at^1^ (accessed on 25 November 2022). Isolates showing inhibition zones equal or above the CO_WT_ value were considered WT, which indicates an absence of acquired resistance mechanisms with phenotypic expression to the tested antimicrobials. Isolates with inhibition zones below the CO_WT_ are designated non-wild type (NWT) and assumed to have acquired a resistance mechanism with phenotypic expression (European Committee on Antimicrobial Susceptibility Testing (EUCAST), 2021).

### 2.3. Screening of resistance determinants

The presence of *mecA* and *blaZ* genes (resistance to beta-lactams) was tested by PCR for all isolates. Other resistance genes were screened only for resistant and intermediate isolates, namely: *erm*(A), *erm*(B), *erm*(C), *vga*(C) (resistance to macrolides and lincosamides); *aadD*, *aph3-IIIa*, *aacA-aphD* (resistance to aminoglycosides), *cat*_pC221_ (resistance to chloramphenicol); *tet*(K), *tet*(M), *tet*(L) (resistance to tetracyclines); *dfrA*(S1), *dfr*(G) (resistance to trimethoprim-sulfamethoxazole) and *fusB*, *fusC* (resistance to fusidic acid). The primers used are detailed in Supplementary Table 2.

To characterize mutations in the quinolone-resistance determining regions (QRDR) of the fluoroquinolone target genes *grlA* and *gyrA*, partial internal fragments of both genes were amplified by PCR as described previously (Costa et al., 2021). The resulting amplicons were purified using the Kit NZYGelpure (NZYTech) and sequenced. Nucleotide sequences were examined with SnapGeneViewer version 5.1.4 (Insightful Science; available at snapgene.com). The corresponding polypeptide sequences were aligned with MEGA X version 10.2.4 (available at^2^), using the GrlA and GyrA sequences of the reference strain *S. pseudintermedius* HKU 10–03 (GenBank accession numbers ADV06974.1 and ADV05612.1, respectively). The primers used are described in Supplementary Table 2.

### 2.4. Molecular typing by PFGE and MLST

The collection was previously characterized by *agr*-typing (Andrade et al., 2022). All 155 *S. pseudintermedius* isolates were typed by macrorestriction analysis with *Sma*I using well-established protocols. The agarose disks with intact chromosomal DNA were prepared following the recommendations for *S. aureus* (Chung et al., 2000) and restricted with *Sma*I (New England Biolabs, Ipswich, MA, United States) according to the manufacturer’s instructions. The restriction fragments were resolved by PFGE with a contour-clamped homogeneous electric field apparatus (CHEF-DRIII, Bio-Rad) in a 1% (w/v) agarose gel using pulse time at 2 s followed by 5 s at 5.6 V/cm for 24 h (Paul et al., 2012). Lambda ladder DNA (New England Biolabs) was used as a molecular weight marker and DNA disks of *S. aureus* NCTC8325 were used as reference. The *Sma*I-macrorestriction profiles of genomic DNA was analyzed with Bionumerics software v.8.0 (Applied Maths, Kortrijk, Belgium). The dendrogram was constructed with DICE and UPGMA algorithms, using a band position tolerance of 1.5% and an optimization of 0.5%. PFGE types presenting ≥ 93% similarity and with the same *agr*-type were considered as part of the same PFGE type (Paul et al., 2012; Ventrella et al., 2017). The genetic diversity of the collection was determined with the Simpson’s Diversity Index (SDI) based on PFGE types and with a confidence interval of 95% (Carriço et al., 2006).

Multilocus sequence typing (MLST) was carried out for 68 isolates (43 MRSP, 25 MSSP), selected according to their PFGE type, antimicrobial resistance profile and *agr*-type. This method was performed by amplification and sequencing of internal fragments of seven housekeeping genes (*ack*, *cpn*60, *fdh*, *pta*, *purA*, *sar*, *tuf*) (Solyman et al., 2013). Nucleotide sequences were analyzed with SnapGeneViewer version 5.1.4 and submitted to the *S. pseudintermedius* MLST database^3^ to retrieve allelic profiles and corresponding ST. New alleles and ST profiles were submitted to PubMLST for validation and allele/ST assignment. Isolates within the same PFGE type were considered as belonging to the same ST. Phylogenetic relations with other STs described for *S. pseudintermedius* were evaluated with the web-based tool PHYLOViZ online.^4^ Due to limited data in literature, the assignment of clonal complexes (CC) was performed with the aid of PHYLOViZ, taking into consideration all the STs described in our collection as well as all the STs described for *S. pseudintermedius* in the PubMLST database until November 2022. The CCs were defined by STs that share at least six identical alleles (SLVs) and were assigned when including at least five STs.

### 2.5. Statistical analysis

Statistical analyses were performed with SPSS v26.0 (IBM Corp.) to evaluate differences between MRSP and MSSP resistance patterns using the chi-square test and the Fisher’s Exact Test. Statistical significance was considered for values of *p* < 0.05.

## 3. Results

### 3.1. Antimicrobial susceptibility profiles

#### 3.1.1. Antimicrobials with established clinical breakpoints

Forty-eight out of the 155 *S. pseudintermedius* isolates (48/155, 31.0%) carried the *mecA* gene and were classified as MRSP. Three of these *mecA*^+^ isolates showed oxacillin zones of inhibition of 19, 21 and 24 mm, which correspond to susceptibility according to CLSI (S ≥ 18 mm; Clinical and Laboratory Standards Institute (VET01S CLSI), 2020; Clinical and Laboratory Standards Institute (CLSI), 2022). Oxacillin MIC determination classified the isolate with a zone of inhibition of 19 mm as intermediate (oxacillin MIC of 0.75 mg/l), while the other two isolates remained categorized as susceptible (oxacillin MICs ≤0.19 mg/l; Clinical and Laboratory Standards Institute (VET01S CLSI), 2020; Clinical and Laboratory Standards Institute (CLSI), 2022).

Of the 155 *S. pseudintermedius*, only 19 (19/155, 12.3%) were susceptible to all antimicrobials tested. Thirty-one (31/155, 20.0%) and 35 isolates (35/155, 22.6%) were resistant to at least one or two antimicrobials from distinct classes, respectively. Finally, 70 isolates (70/155, 45.2%) presented an MDR profile. The antimicrobial resistance profiles found in the collection are presented in Supplementary Figure 1; Table 1.

**Table 1:** Distribution of antimicrobial resistance phenotypes among the 155  *S. pseudintermedius* studied and associated acquired resistance gene(s).

Antimicrobials	Resistance phenotype frequency (*n*) (%)			Associated acquired resistance gene(s)	
	Intermediate		Resistant	Gene	Frequency [(*n*/*N*) %]
Beta-lactams					
PEN	NA		132 (85.2)	*bla*Z	84/155 (54.2)
				*bla*Z + *mecA*	48/155 (31.0)
OXA	NA		46 (29.7)	*mecA*	48/155 (31.0)
Tetracyclines					
TET	1 (0.6)		85 (54.8)	*tet*(M)	57/86 (66.3)
				*tet*(K)	20/86 (23.3)
				*tet*(M) + *tet*(K)	8/86 (9.3)
				not identified	1/86 (1.2)
TET + MIN	24 (15.5)		1 (0.6)	*tet*(M)	21/25 (84.0)
				*tet*(M) + *tet*(K)	3/25 (12.0)
				*tet(M)* + *tet*(L)	1/25 (4.0)
Aminoglycosides					
GEN	4 (2.6)		35 (22.6)	*aph3-IIIa* + *aacA-aphD*	33/39 (84.6)
				*aacA-aphD*	4/39 (10.3)
				*aph3-IIIa*	1/39 (2.6)
				Not identified	1/39 (2.6)
Macrolides and Lincosamides					
ERY + CLI	0		49 (31.6)	*erm*(B)	45/49 (91.8)
				*erm*(B) + *erm*(C)	2/49 (4.1)
				Not identified	2/49 (4.1)
ERY + CLI_i_	0		8 (5.2)	*erm*(B)	8/8 (100)
SXT	1(0.6)		46 (29.7)	*dfr*(G)	46/47 (97.9)
				Not identified	1/47 (2.1)
CHL	0		24 (15.5)	*catp*_C221_	22/24 (91.7)
				Not identified	2/24 (8.3)
FUS	0		7 (4.5)	*fus*(C)	1/7 (14.3)
				Not identified	6/7 (85.7)
RIF	2 (1.3)		1 (0.6)	ND	NA
Fluoroquinolones					
*Please see * Figure 1					

Susceptibility profiles were interpreted according to: VET01S (Clinical and Laboratory Standards Institute (VET01S CLSI), 2020) (CLI, TET), M100-ED32 (Clinical and Laboratory Standards Institute (CLSI), 2022) (PEN, OXA, GEN, ERY, MIN, SXT, CHL, RIF) and European Committee on Antimicrobial Susceptibility Testing (EUCAST) (2023) (FUS).

The *blaZ* and *mecA* genes were screened for the entire collection; the remaining determinants were tested for resistant or intermediate isolates.

PEN: penicillin; OXA: oxacillin; TET: tetracycline; MIN: minocycline; GEN: gentamycin; ERY: erythromycin; CLI: clindamycin; CLI_i_: inducible clindamycin resistance; SXT: trimethoprim-sulfamethoxazole; CHL: chloramphenicol; FUS: fusidic acid; RIF: rifampicin; CIP: ciprofloxacin; MOX: moxifloxacin; ENR: enrofloxacin; PRA: pradofloxacin; NA: not applicable; ND: not determined.

Resistance was frequently detected toward penicillin (132/155, 85.2%), tetracycline (86/155, 55.5%), erythromycin (57/155, 36.8%), clindamycin (57/155, 36.8%), trimethoprim-sulfamethoxazole (47/155, 30.3%), gentamycin (39/155, 25.2%) and fluoroquinolones (39/155, 25.2%). Resistant isolates were also detected for minocycline (25/155, 16.1%), chloramphenicol (24/155, 15.5%) and rifampicin (3/155, 1.9%). We also observed resistance to fusidic acid (7/155, 4.5%). All isolates were susceptible to linezolid, tigecycline and quinupristin–dalfopristin. Within the entire collection, 43 different resistance patterns were observed, of which resistance to penicillin and tetracycline was the most frequent (30/155, 19.4%).

**Figure 2:**
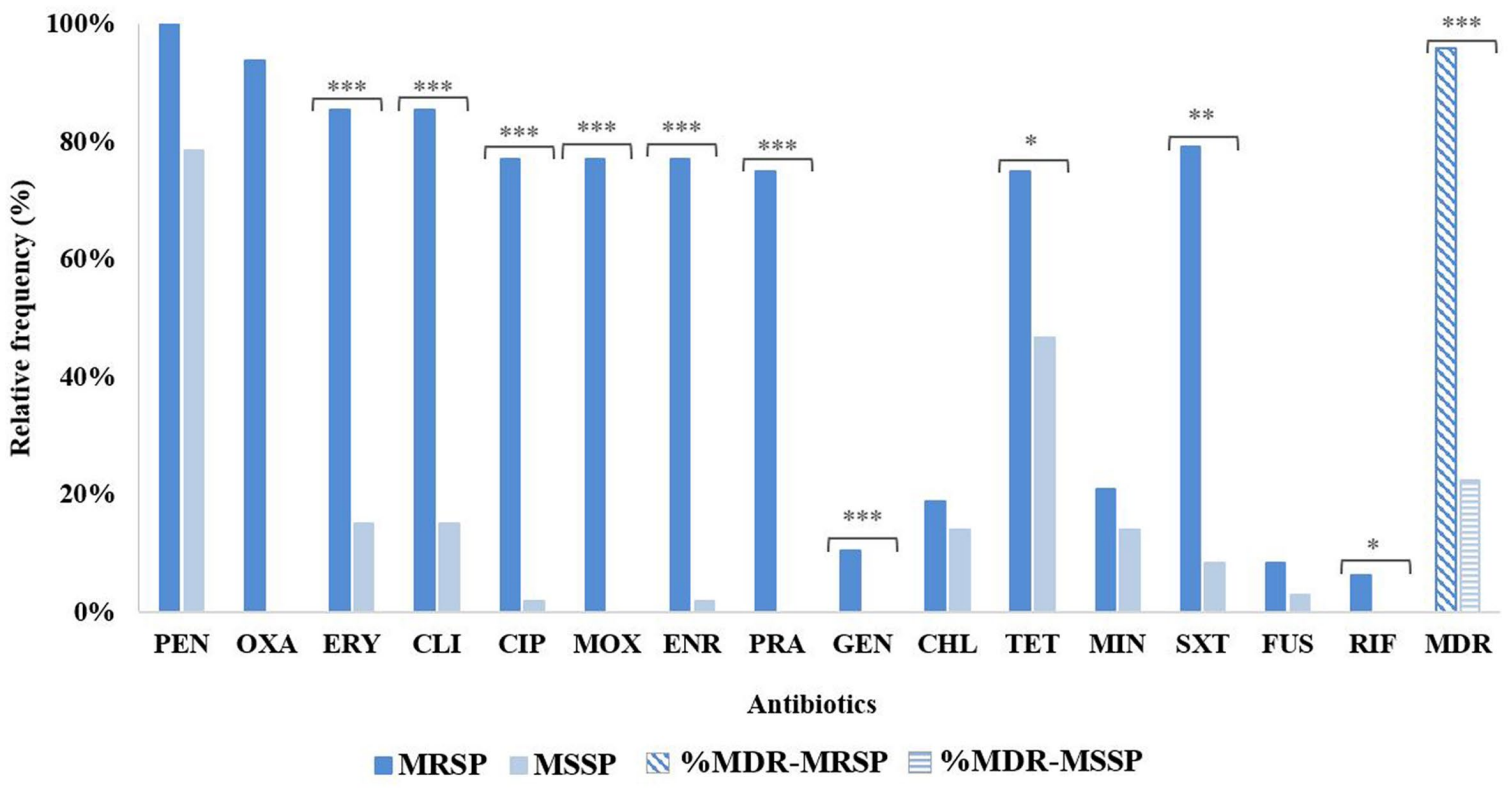
Distribution of antimicrobial resistance profiles for MRSP (*n* = 48) and MSSP (*n* = 107), including MDR frequency. PEN, penicillin; OXA, oxacillin; ERY, erythromycin; CLI, clindamycin; CIP, ciprofloxacin; MOX, moxifloxacin; ENR, enrofloxacin; PRA, pradofloxacin; GEN, gentamycin; CHL, chloramphenicol; TET, tetracycline; MIN, minocycline; SXT, trimethoprim-sulfamethoxazole; FUS, fusidic acid; RIF, rifampicin; MDR, multidrug resistance. Statistical differences are represented as follows: ***p < 0.00001; **p < 0.0001; *p < 0.05.

Among the 48 MRSP, 46 had an MDR profile (46/48, 95.8%), whereas for MSSP the MDR frequency was 22.4% (24/107), a difference statistically significant (*p* < 0.00001; Figure 2). Other statistically significant differences between MRSP and MSSP corresponded to the number of resistant isolates for fluoroquinolones (*p* < 0.00001), macrolides/lincosamides (*p* < 0.00001), aminoglycosides (*p* < 0.00001), trimethoprim-sulfamethoxazole (*p* < 0.0001), tetracycline (*p* = 0.001058) and rifampicin (*p* = 0.0284). No significant differences were observed for chloramphenicol, minocycline and fusidic acid (Figure 2). Among MRSP-MDR, the most common resistance pattern (14/46, 30.4%) included resistance to beta-lactams, erythromycin, clindamycin, fluoroquinolones, gentamycin, tetracyclines, and trimethoprim-sulfamethoxazole. For MSSP, the two most common MDR phenotypes included resistance to penicillin, erythromycin, clindamycin and chloramphenicol (5/24, 20.8%); or to penicillin, tetracycline and trimethoprim-sulfamethoxazole (5/24, 20.8%).

#### 3.1.2. Antimicrobials without established clinical breakpoints

Regarding the nine antimicrobials without breakpoints available at either CLSI or EUCAST, the distributions of the zones of inhibition for each antimicrobial were used to estimate a cut-off (CO_WT_) value, according to the Normalized Resistance Interpretation method (Table 2). The distributions of the zones of inhibition are presented in Supplementary Figure 2. NWT populations were detected at higher frequencies for the aminoglycosides kanamycin, neomycin and tobramycin (varying between 28.4–41.9%) and at lower frequencies for bacitracin, novobiocin, mupirocin, florfenicol and amikacin (1.9–7.7%). No NWT isolates were identified for apramycin.

**Table 2:** Cut-off (CO_WT_) values of the 155 *S. pseudintermedius* for antimicrobials without available breakpoints, determined based on the distribution of the zones of inhibition (mm), according to the normalized resistance interpretation method.

Antimicrobials	CO_WT_ (mm)	SD (mm)	WT Population (no. isolates) (%)	NWT Population (no. isolates) (%)	Associated acquired resistance gene(s)	
					Gene	Frequency (n/N) (%)
AMK	21	1.47	152 (98.1)	3 (1.9)	*aph3-IIIa* + *aacA-aphD*	3/3 (100)
APR	18	1.60	155 (100)	0 (0)	–	–
KAN	20	1.59	90 (58.1)	65 (41.9)	*aph3-IIIa* + *aacA-aphD*	36/65 (55.4)
					*aph3-IIIa*	23/65 (35.4)
					*aacA-aphD*	5/65 (7.7)
					Not identified	1/65 (1.5)
NEO	19	1.32	95 (61.3)	60 (38.7)	*aph3-IIIa* + *aacA-aphD*	36/60 (60)
					*aph3-IIIa*	23/60 (38.3)
					Not identified	1/60 (1.7)
TOB	22	1.32	111 (71.6)	44 (28.4)	*aph3-IIIa* + *aacA-aphD*	36/44 (81.8)
					*aacA-aphD*	5/44 (11.4)
					*aph3-IIIa*	1/44 (2.3)
					Not identified	2/44 (4.5)
NOV	33	2.03	145 (93.5)	10 (6.5)	ND	ND
FFC	24	1.78	152 (98.1)	3 (1.9)	ND	ND
MUP	33	2.46	151 (97.4)	4 (2.6)	ND	ND
BAC	12	1.23	143 (92.3)	12 (7.7)	ND	ND

CO_WT_, cut-off value; SD, standard deviation; WT, wild-type; NWT, non wild-type; AMK, amikacin; APR, apramycin; KAN, kanamycin; NEO, neomycin; TOB, tobramycin; NOV, novobiocin; FFC, florfenicol; MUP, mupirocin; BAC, bacitracin; ND, not determined.

### 3.2. Relation between acquired resistance genes and antimicrobial susceptibility profiles

Carriage of acquired resistance genes was screened for all isolates categorized either as resistant, intermediate or for most of the assigned to NWT populations.

Regarding beta-lactam resistance, a good correlation was observed between resistance phenotype and presence of resistance genes. All *S. pseudintermedius* resistant to oxacillin and penicillin carried the *mecA* or *blaZ* and/or *mecA* genes, respectively (Table 1) except for two *mecA*^+^ isolates categorized as susceptible to oxacillin, as described above. All but one of the 86 isolates resistant to tetracycline carried *tet* determinants, either alone or in different combinations (Table 1). Two thirds (65/86, 75.6%) of the isolates carried the *tet*(M) gene, and 25 of them were resistant to minocycline. In addition, 28 isolates (28/86, 32.6%) presented the *tet*(K) gene, eight of them simultaneously with *tet*(M). Only one isolate, with resistance to both tetracyclines, carried the *tet*(L) gene in combination with *tet*(M).

Co-carriage of *aacA-aphD* and *aph3-IIIa* genes (36/65, 55.4%) was detected in most isolates resistant to gentamycin or NWT for kanamycin, neomycin, tobramycin and amikacin. Single carriage of *aacA-aphD* (5/65, 7.7%) or *aph3-IIIa* (23/65, 35.4%) was also associated with those phenotypes. The gene *aadD* was screened but not detected among the collection. One isolate resistant to gentamycin (and NWT to tobramycin and kanamycin) carried none of the genes screened.

Resistance to erythromycin and clindamycin was related to carriage of the *erm*(B) gene for 53 out of the 57 resistant isolates (53/57, 93.0%) and associated with co-carriage of *erm*(B) and *erm*(C) genes for other two isolates (2/57, 3.5%). The remaining genes searched (*vga*(C) and *erm*(A)) were not detected and two isolates did not carry any of the genes screened.

All but one of the isolates resistant to trimethoprim-sulfamethoxazole carried the *dfr*(G) gene (46/47, 97.9%), whereas the *dfrA*(S1) gene was not detected. The remaining isolate did not carry any of these genes.

Resistance to chloramphenicol was related to the *cat*_pC221_ gene for the majority of the resistant isolates (22/24, 91.7%). Regarding fusidic acid, an association with a resistance gene was only obtained for a single isolate, carrying *fusC*.

### 3.3. Fluoroquinolone resistance and QRDR mutations

**Figure 1:**
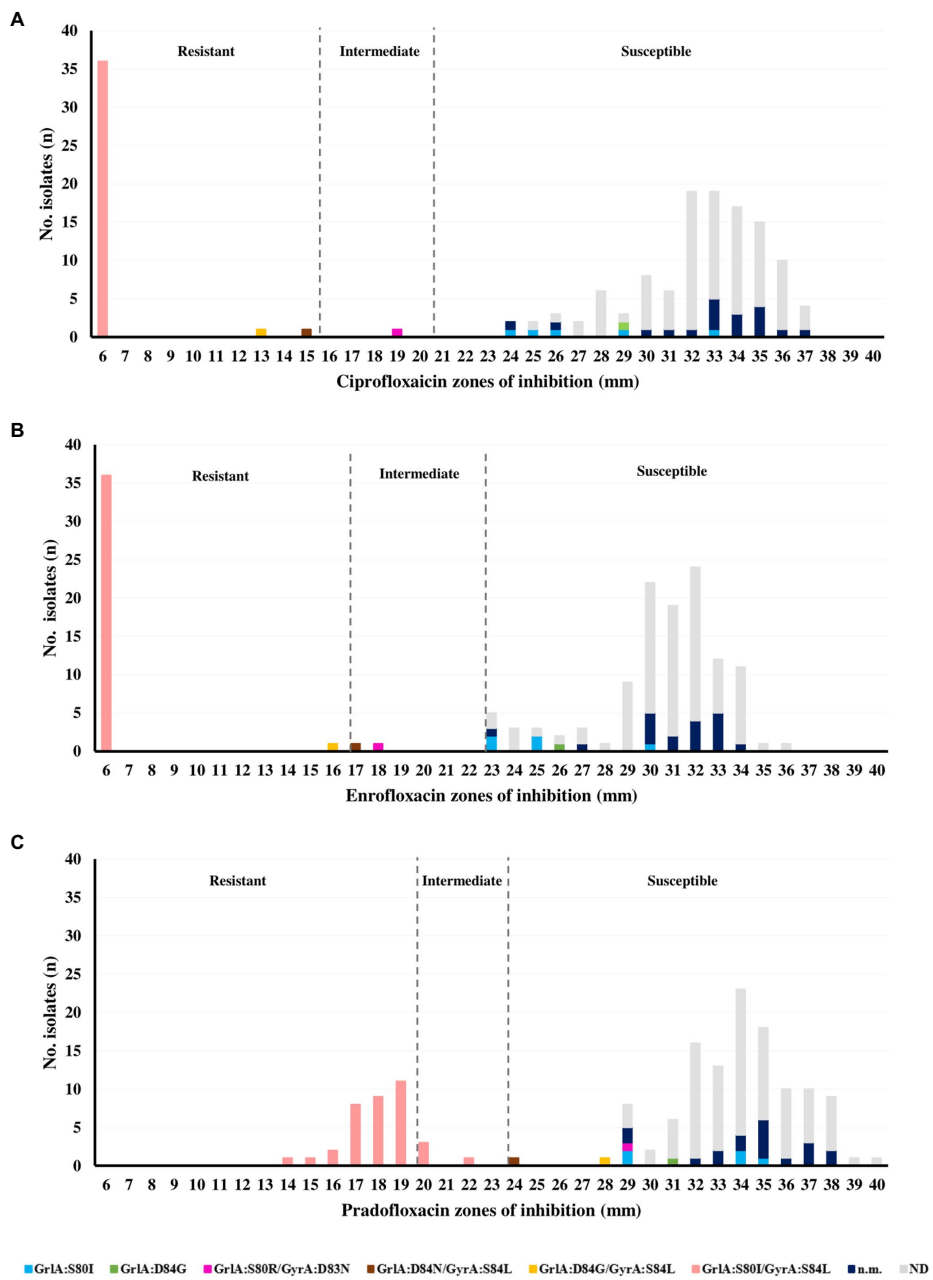
Relation between the zones of inhibition and QRDR mutations obtained for **(A)** ciprofloxacin, **(B)** enrofloxacin, and **(C)** pradofloxacin in the *Staphylococcus pseudintermedius* collection (*n* = 155).

A quarter of the *S. pseudintermedius* (39/155, 25.2%) collection was resistant to fluoroquinolones. In particular, 39, 37 and 36 isolates (25.2, 23.9 and 23.2%) were resistant to enrofloxacin and ciprofloxacin, moxifloxacin or pradofloxacin, respectively (Supplementary Figure 1; Figure 1).

The main mechanism of resistance to fluoroquinolones in *S. pseudintermedius* is the occurrence of mutations in the QRDR regions of the target GrlA and GyrA proteins. The distribution of the zones of inhibition for ciprofloxacin, enrofloxacin and pradofloxacin (the last two of exclusive veterinary use) are presented in Figure 1, together with the six different patterns of QRDR mutations detected. The most frequent mutation pattern was the double mutation GrlA:S80I/GyrA:S84L (36/39, 92.3%) linked to a resistance phenotype toward ciprofloxacin, enrofloxacin and pradofloxacin. To our knowledge, the mutation GyrA:D83N is here described for the first time in *S. pseudintermedius*, occurring in combination with GrlA:S80R in an isolate with intermediate phenotype for both enrofloxacin and ciprofloxacin. All isolates with a single GrlA mutation (S80I or D84G) were categorized as susceptible to all fluoroquinolones tested, according to the disk diffusion results (Figure 1).

### 3.4. Genetic diversity of the *Staphylococcus pseudintermedius* collection

#### 3.4.1. PFGE typing

**Figure 3:**
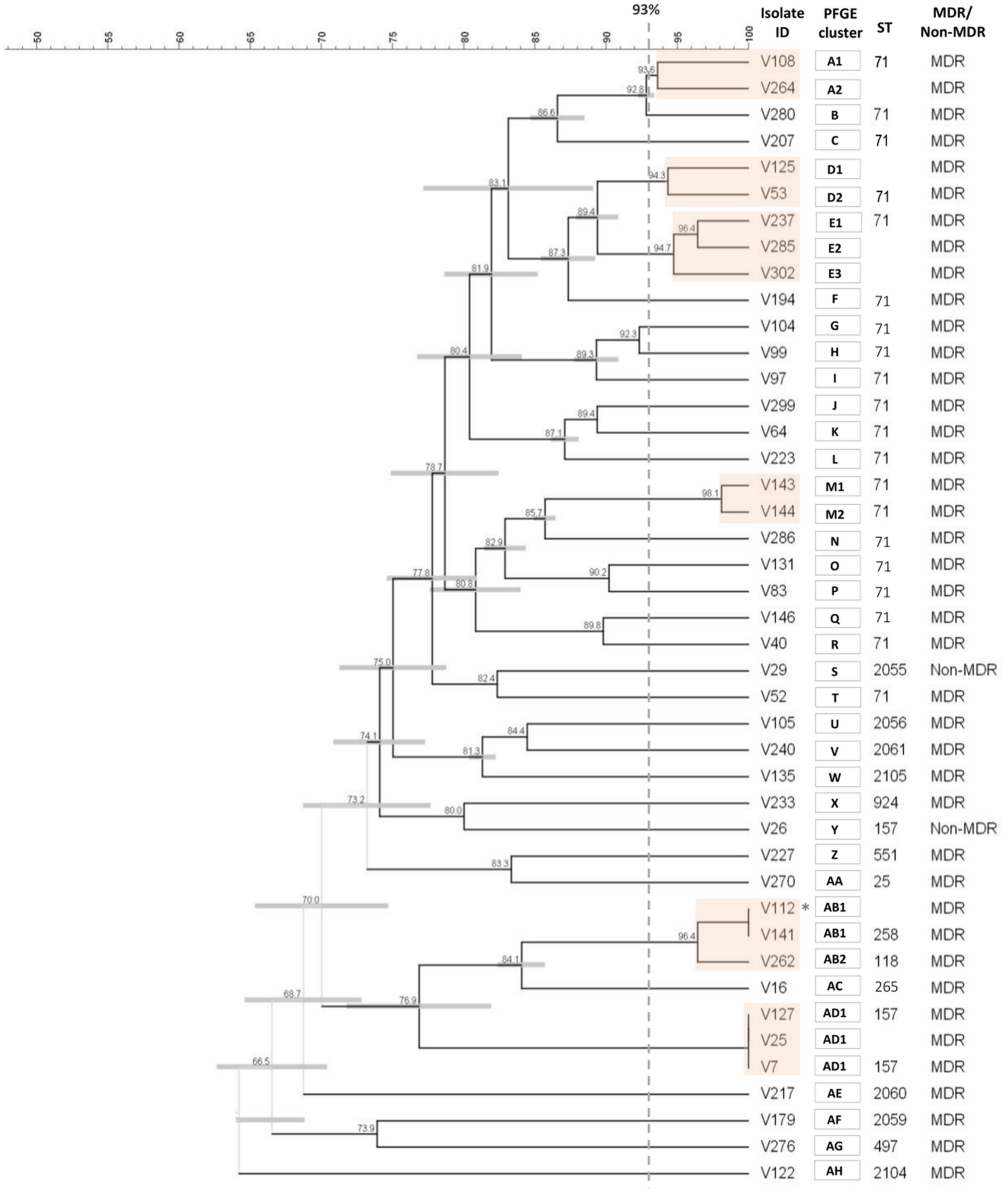
Cluster analysis of the *Sma*I macrorestriction profiles of the 43 MRSP isolates. The dendrogram was constructed with DICE and UPGMA algorithms using an optimization of 0.5% and tolerance of 1.5%. PFGE types (orange boxes) were defined by a cut-off value ≥93% of similarity (gray dashed line). Each type is designed by capital letter(s), with numbers indicating subtypes. * Isolates V112 and V141 were collected from the same animal. Because they were pheno- and genotypically indistinguishable; isolate V112 was not included in the final analysis.

PFGE typing was performed for the entire collection. Six out of the 155 *S. pseudintermedius* isolates were non-typable, which corresponded to five MRSP and one MSSP isolate. The remaining 149 *S. pseudintermedius* were distributed within 129 PFGE types (A-DY). This corresponds to an SDI of 0.9979 (95% confidence interval of 0.9964–0.9993), which indicates a high genetic diversity for the collection. The 43 MRSP were distributed in 34 PFGE types (A-AH; Figure 3; Supplementary Figure 3), whereas the 106 MSSP corresponded to 95 PFGE types (data not shown).

#### 3.4.2. MLST typing

**Figure 4:**
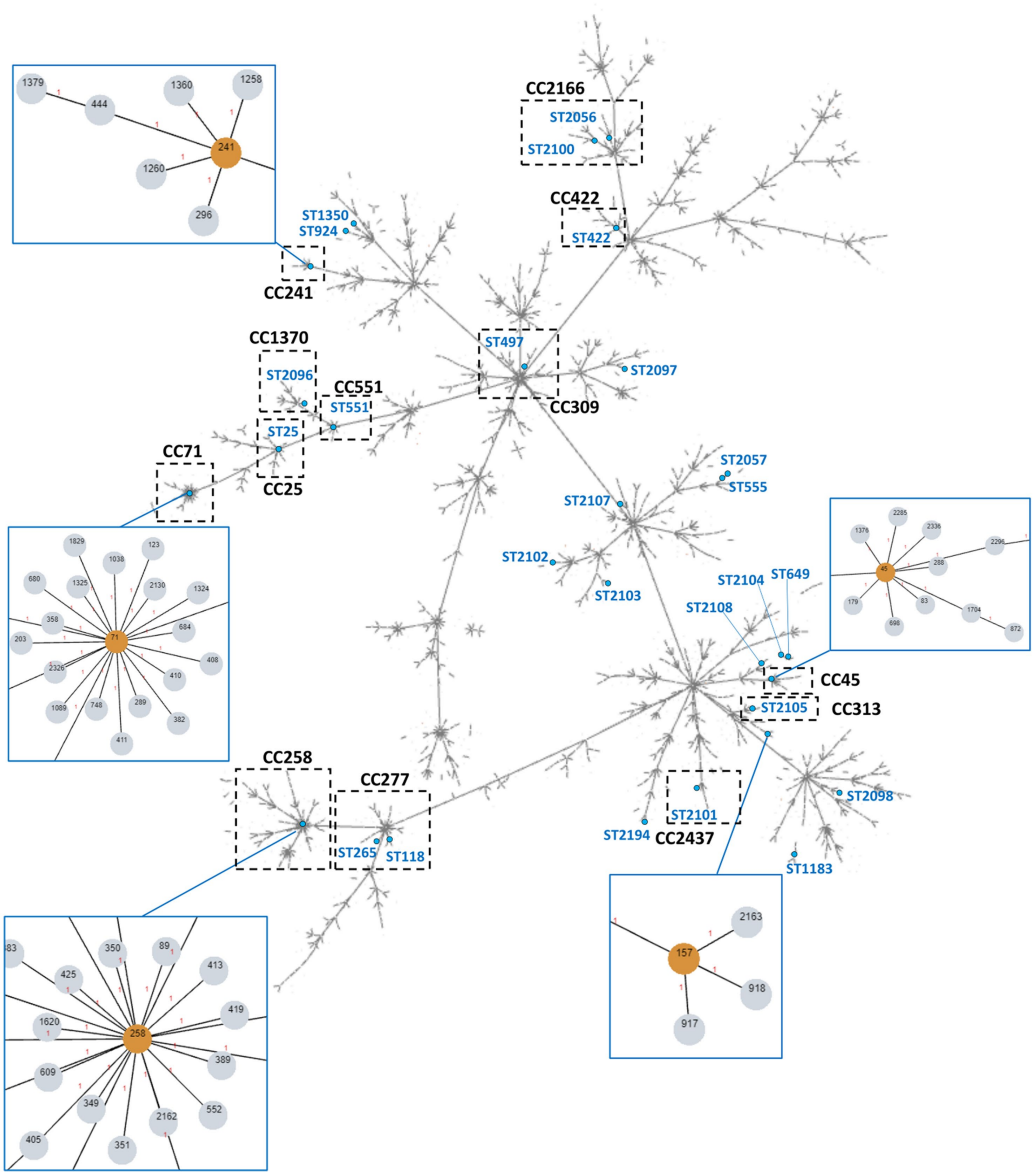
Overview of the global *S. pseudintermedius* population clonal structure. The tree was built using PHYLOViZ online with all *S. pseudintermedius* STs described in PubMLST database until November 2022, linking all SLVs. The STs found in the collection in study are highlighted in blue lettering or singled out in orange within the blue boxes. The assigned clonal complexes are indicated in bold-type lettering. Dashed boxes highlight CCs containing STs identified in this work. Some of these CCs are zoomed in blue boxes for higher resolution.

MLST was performed for 68 isolates (43 MRSP, 25 MSSP), representing the main PFGE types or resistance phenotypes found in the study collection. A total of 42 STs were identified among the 68 isolates typed (Table 3; Figure 4), again confirming the high diversity of the collection. Twenty-five STs (ST2054-ST2061; ST2095-ST2109; ST2194-ST2195) were described for the first time in this study, identified mainly among MSSP isolates (17/25). For the remaining analysis, we assumed that isolates grouped within the same PFGE type and sharing the same *agr*-type belong to the same lineage (same ST). The lineage most frequently detected was ST71 (24/155, 15.5%), exclusively associated with MRSP-MDR isolates. Other lineages detected in two or more isolates were ST157 (*n* = 4), ST45 (*n* = 3), ST241 (*n* = 3), ST2054 (*n* = 2), ST2061 (*n* = 2), and ST2194 (*n* = 2). Sequence types represented by a single isolate include ST258, ST265, and ST551, which are emerging clonal lineages in Europe. To the best of our knowledge, this is the first report of ST258 in Portugal.

**Table 3:** Phenotypic and molecular characteristics of the MRSP and MSSP isolates typed by MLST.

CC	ST	Allelic profile							MRSPMSSP	*n*	Resistance phenotype^(^^a^^)^										Resistance genotype^(^^b^^)^											
		*ack*	*cpn60*	*fdh*	*pta*	*purA*	*sar*	*tuf*			PEN	OXA	ML	AG	FQ	TET	SXT	CHL	FUS	RIF	*blaZ*	*mecA*	*erm*(B)	*erm*(C)	*aph3-IIIa*	*aacA-aphD*	*tet*(M)	*tet*(L)	*tet*(K)	*dfr*(G)	*cat*_pC221_	*fus*(C)
25	25	3	9	2	1	1	1	1	MRSP	1																						
45	45	3	2	2	1	2	1	2	MRSP	2																						
										1																						
71	71	3	9	1	2	1	2	1	MRSP	13																						
										1																						
										5																						
										2																						
										1																						
										1																						
										1																						
277	118	5	13	8	1	11	1	2	MRSP	1																						
	265	5	13	4	1	3	1	2	MRSP	1																						
	157	2	11	2	11	5	1	2	MRSP	1																						
										1																						
										1																						
										1																						
258	258	5	13	4	1	11	2	2	MRSP	1																						
422	422	3	2	2	1	1	1	2	MRSP	1																						
309	497	1	11	2	1	1	1	1	MRSP	1																						
551	551	5	9	2	1	1	1	1	MRSP	1																						
	924	1	11	4	1	20	1	1	MRSP	1																						
313	2105	5	21	2	1	5	1	2	MRSP	1																						
	2055	5	8	2	1	18	2	1	MRSP	1																						
	2057	3	8	4	36	11	1	1	MRSP	1																						
	2059	4	13	1	23	10	2	5	MRSP	1																						
	2060	5	24	2	36	11	1	1	MRSP	1																						
	2061	5	24	1	1	5	5	1	MRSP	2																						
	2104	3	3	33	4	11	1	2	MRSP	1																						
2166	2056	1	9	2	1	5	1	2	MRSP	1																						
	2100	1	9	2	1	7	1	2	MSSP	1																						
241	241	1	21	4	1	23	1	1	MSSP	2																						
										1																						
	455	1	2	2	1	20	19	2	MSSP	1																						
	555	3	8	4	1	11	1	1	MSSP	1																						
	649	3	3	5	4	11	1	2	MSSP	1																						
	1183	5	7	7	1	3	1	1	MSSP	1																						
	1350	1	6	4	1	3	1	1	MSSP	1																						
	2054	9	6	2	23	8	1	1	MSSP	2																						
	2058	3	6	11	1	7	1	2	MSSP	1																						
	2095	64	110	2	1	14	1	1	MSSP	1																						
1370	2096	3	8	4	1	1	1	1	MSSP	1																						
	2097	1	18	1	1	3	1	1	MSSP	1																						
	2098	2	25	2	4	11	1	2	MSSP	1																						
	2099	4	9	4	2	11	1	1	MSSP	1																						
2437	2101	5	24	2	1	10	1	2	MSSP	1																						
	2102	5	2	3	1	8	1	1	MSSP	1																						
	2103	5	6	2	1	3	3	1	MSSP	1																						
	2106	5	8	3	36	1	2	1	MSSP	1																						
	2107	1	9	2	4	11	1	1	MSSP	1																						
	2108	3	2	2	4	7	1	2	MSSP	1																						
	2109	5	21	2	36	2	2	1	MSSP	1																						
	2194	1	113	2	2	3	2	2	MSSP	2																						
	2195	2	6	4	4	11	1	2	MSSP	1																						

(a) resistance/intermediate (dark blue)/susceptible (light blue).

(b) acquired resistance gene (gray)/absent (white); PEN, penicillin; OXA, oxacillin; ML, macrolides/lincosamides; AG, aminoglycosides; FQ, fluoroquinolones; TET, tetracyclines; SXT, trimethoprim-sulfamethoxazole; CHL, chloramphenicol; FUS, fusidic acid; RIF, rifampicin.

PHYLOViZ analysis of our MLST data together with the all STs available at PubMLST (until November 2022) showed that the STs detected in our *S. pseudintermedius* collection were distributed over 13 clonal complexes, with CC71, CC45 and CC241 as the most frequent CCs (Table 3; Figure 4). Nineteen STs did not belong to an assigned CC and 14 STs corresponded to singletons.

## 4. Discussion

*S. pseudintermedius* is the main bacterial agent of canine pyoderma (Lynch and Helbig, 2021) and this skin infection is one of the main reasons for antimicrobial prescription in companion animals (Loeffler and Lloyd, 2018). Three studies have evaluated the resistance trend of *S*. *pseudintermedius* causing several infections in Portugal (Couto et al., 2014, 2016; Silva et al., 2021). However, none of them analyzed the genetic diversity in *S. pseudintermedius* obtained exclusively from skin infections nor included both MSSP and MRSP strains.

### 4.1. Overview of the antimicrobial resistance burden

The *S. pseudintermedius* collection was characterized by a high burden of antimicrobial resistance, with a considerable proportion of MDR and MRSP strains (45.2 and 31.0%, respectively). The MDR phenotype was statistically linked to MRSP strains (*p* < 0.00001). Our data supports the increasing trend of antimicrobial resistance in *S. pseudintermedius* reported throughout Europe (Haenni et al., 2014; Grönthal et al., 2017; Ventrella et al., 2017; Nocera et al., 2021; Ruiz-Ripa et al., 2021) and also in Portugal. In particular, previous data from the Lisbon area, where our study focused, already reported increasing trends of antimicrobial resistance, with overall prevalence of 39.0% MDR and 8.7% of MRSP for the 1999–2014 period (Couto et al., 2016). More recently, a study with 31 MRSP strains showed that all of these had an MDR phenotype (Silva et al., 2021).

In our collection, MDR patterns exhibited by MRSP strains often included resistance to at least five antimicrobial classes other than beta-lactams (Table 3). These results confirm the resistance build-up among MRSP in our country, as reported by Silva et al. (2021). Similar findings have been described for MRSP from Spain (Viñes et al., 2022). The fewer MDR phenotypes displayed by MSSP strains were more variable, in accordance with data from other European countries (Haenni et al., 2020), and included mostly resistance to up to four antimicrobial classes (Figure 2).

The recommended first-line systemic treatment for canine pyoderma is based on the use of beta-lactams, clindamycin and trimethoprim-sulfamethoxazole, whereas tetracyclines, aminoglycosides and fluoroquinolones are recommended as second-line therapeutics (Hillier et al., 2014; Morris et al., 2017). Fusidic acid is recommended for the treatment of human infections caused by methicillin-resistant *S. aureus* and for localized infections in canine pyoderma. However, in companion animals this antimicrobial should be reserved for infections caused by isolates resistant to other topical antimicrobials and the treatment could be complemented, when necessary, by systemic antimicrobials (Hillier et al., 2014; Morris et al., 2017). The available prescribing data in Europe reflect therapeutic divergences between countries. In the United Kingdom, the most frequently prescribed antimicrobials for canine infections, including pyoderma, were amoxicillin-clavulanate, cefalexin, cefovecin, clindamycin and fluoroquinolones (Summers et al., 2014; Singleton et al., 2017). The same antimicrobials, except cefovecin, were frequently prescribed in Italy, for skin infections (Chirollo et al., 2021). In Portugal (Oliveira et al., 2018,) and Spain (Madrid) (Gómez-Poveda and Moreno, 2018), the most prescribed antimicrobials for treatment of skin infections in companion animals were amoxicillin-clavulanate, cefalexin and fluoroquinolones.

A striking and worrisome observation from the analysis of the antibiotic susceptibility profiling of our *S. pseudintermedius* is the continuing build-up of resistance to all major first- and second-line antimicrobials important for systemic SSTIs management in Europe, confirming the increasing trend previously observed by Couto et al. (2016), and which could reflect the consumption of such antimicrobials for companion animals over the years in Portugal. Although that earlier work included *S. pseudintermedius* causing pyoderma and other infections (mostly otitis and urinary tract infections), comparison between the two studies highlights a nearly three-fold increase in resistance frequency rate for fluoroquinolones (9.2% vs. 25.2%), and nearly a two-fold increase for trimethoprim-sulfamethoxazole (16.4% vs. 30.3%), aminoglycosides (23.3% vs. 41.9%) and clindamycin (20.6% vs. 36.8%). Although in a lesser extent, increase in resistance to penicillin (64.4% vs. 85.2%) and tetracyclines (44.0% vs. 55.5%) is also relevant, as reported for other European countries (Haenni et al., 2020), including Spain (Ruiz-Ripa et al., 2021; Viñes et al., 2022), and Italy (Bellato et al., 2022). Resistance to fusidic acid (4.5%) followed the same increasing trend when compared to previous data from Portugal [0.9% (Couto et al., 2016) or 3.2% (Silva et al., 2021)].

One of the limitations while studying antimicrobial resistance in bacteria isolated from animals is the lack of available breakpoints for all antimicrobials. To obviate this limitation, we estimated CO_WT_ values for nine antimicrobials for which there are no established breakpoints either by CLSI or EUCAST (Table 2; Supplementary Figure 2). In our study, the application of the estimated CO_WT_ allowed to detect NWT populations for aminoglycosides that carried *aacA-aphD* or the *aph3-IIIa* determinants. Another example of the applicability of the CO_WT_ parameter is illustrated by mupirocin, a last-resort topical antimicrobial recommended for pyoderma treatment and proposed for *S. pseudintermedius* decolonization protocols (Cuny et al., 2022), for which four NWT isolates were detected. While the absence of a clinical breakpoint may hinder the detection of these isolates, potentially carrying resistance determinants, it is expected that the use and complementation of these CO_WT_ values by other groups and researchers may assist on their refinement for the rapid identification of NWT populations and prevent potential therapeutic failure.

Even for antimicrobials with established breakpoints, there is a need for continuous update. This may be illustrated by the three *S. pseudintermedius mecA^+^* strains, all from new and different clonal lineages, classified as susceptible or intermediate to oxacillin by Clinical and Laboratory Standards Institute (CLSI) (2022) and as susceptible or resistant by European Committee on Antimicrobial Susceptibility Testing (EUCAST) (2023) guidelines (European Committee on Antimicrobial Susceptibility Testing (EUCAST), 2023). A recent study suggested that this discrepancy may be related to the type of SCC*mec* carried (Viñes et al., 2022). Updated information from additional studies may clarify these observations.

### 4.2. High genetic diversity of *Staphylococcus pseudintermedius* in companion animals from Portugal

The study collection displayed high genetic diversity, as established by an SDI of 0.9979 and further confirmed by the high number of STs found, including 25 new STs, mostly associated with MSSP strains. The highly diverse nature of *S. pseudintermedius* has been described in other studies (Couto et al., 2014; Pires dos Santos et al., 2016; Gagetti et al., 2019; Haenni et al., 2020), particularly for MSSP. The 25 MSSP characterized were distributed between 23 STs, 17 of them described here for the first time. As for the 48 MRSP, these were assigned to 19 STs, eight of them also new.

Lineage ST71 (CC71) was the most frequently detected, as described in other European countries. This suggests the maintenance of this lineage as the predominant one in our setting, although a recent study suggested a possible replacement of ST71 by ST123 (Silva et al., 2021), an SLV from ST71, already described for MRSP in the Netherlands (Duim et al., 2016). In line with other studies (Pires dos Santos et al., 2016; Worthing et al., 2018; Menandro et al., 2019; Papic et al., 2021; Rynhoud et al., 2021), ST71 was associated only with MRSP and MDR strains, resistant to beta-lactams, macrolides, lincosamides, aminoglycosides, trimethoprim-sulfamethoxazole, fluoroquinolones, and in a lesser extent, to tetracyclines (Table 3). Interestingly, while the first descriptions of CC71 isolates indicate infrequent resistance to tetracycline (Perreten et al., 2010; Videla et al., 2017) recent studies indicate an increase in ST71 strains resistant to this antimicrobial associated with *tet*(K) gene (Worthing et al., 2018; Menandro et al., 2019; Papic et al., 2021; Ruiz-Ripa et al., 2021; Wegener et al., 2021). This is confirmed in our study, with over 70% of ST71 isolates resistant to tetracycline mediated exclusively by *tet*(K) gene.

The second most frequent lineage in our collection was ST157, for which few information is available in the literature. Data from PubMLST indicates that it has been previously described in the USA in an MSSP isolate obtained from a dog (Jolley et al., 2018). Additionally, it was recently reported in a MRSP isolate in Sweden (Swedres-Svarm, 2021). In our study, all four ST157 isolates were MRSP, three of which with an MDR profile (Table 3).

We also detected strains from ST45, a lineage globally disseminated, frequently detected in Asia, but also in Australia, the United States, Canada and Europe (Couto et al., 2016; Pires dos Santos et al., 2016; Grönthal et al., 2017; Worthing et al., 2018; Menandro et al., 2019; Silva et al., 2021; Wegener et al., 2021). It is associated with MRSP and MDR isolates (Pires dos Santos et al., 2016; Worthing et al., 2018; Silva et al., 2021), as observed in our collection.

Our study also identified four important clonal lineages (ST258, ST241, ST551 and ST265) for the first time in Portugal. ST258 belongs to CC258, a clonal complex recently described in the North of Europe, which appears to be replacing CC71 MRSP strains in some European countries (Kizerwetter-Świda et al., 2017). CC258 was previously described in association with both MRSP and MSSP (Pires dos Santos et al., 2016), and to be more susceptible to licensed veterinary antimicrobials, including fluoroquinolones (Pires dos Santos et al., 2016; Bergot et al., 2018). In our study, we detected a ST258 MRSP strain that was resistant to five antimicrobials classes, yet susceptible to fluoroquinolones and aminoglycosides. ST241 was the most frequent clonal lineage among MSSP-MDR in our study, with a profile similar to the one described by Wegener et al. (2021). This clonal lineage was already detected in North of Europe, Spain and France (Lozano et al., 2017; Haenni et al., 2020; Wegener et al., 2021), and isolated in both humans and companion animals (Lozano et al., 2017; Wegener et al., 2021; Glajzner et al., 2022). Lineage ST551 (CC551) was described in the last years in North of Europe and suggested to be replacing CC71 in Poland and Sweden (Kizerwetter-Świda et al., 2017; Swedres-Svarm, 2020, 2021). In our study, this clonal lineage was detected in a MRSP-MDR strain with a resistance profile similar to other ST551 isolates (Swedres-Svarm, 2019; Papic et al., 2021). Our study also describes, for the first time in our country, a MRSP-MDR belonging to ST265 from clonal complex CC277, a lineage frequently detected in the North of Europe (Duim et al., 2016; Pires dos Santos et al., 2016).

These results are relevant to understand the emergence of antimicrobial resistance among *S. pseudintermedius* causing SSTIs in companion animals in Portugal, specifically to antimicrobials used in veterinary and human medicines. Our data reveals high frequency of MRSP strains as well as high frequency of MDR profiles among MRSP and MSSP, which limits the therapy for these infections. ST71 remains the lineage most frequently detected in Portugal, and we report for the first time the introduction in Portugal of several new clonal lineages (ST258, ST241, ST551 and ST265) that have been replacing ST71 in different European regions. The diversity of *S. pseudintermedius* clonal lineages, associated with a high burden of antimicrobial resistance reinforces the need for a correct diagnostic and antimicrobial resistance surveillance to improve the management of these infections and prevent further dissemination of antimicrobial resistance.

## Data availability statement

The original contributions presented in the study are included in the article/Supplementary material, further inquiries can be directed to the corresponding author.

## Ethics statement

Ethical review and approval was not required for the study on animals in accordance with the local legislation and institutional requirements.

## Author contributions

SSC and IC contributed to conception and design of the study. CM, SSC, ML, BR, MA, CF, and IC organized the database. PA performed the statistical analysis. CM, SSC, and IC wrote the first draft of the manuscript. CM, SSC, ML, BR, MA, CF, PA, CP, and IC reviewed and edited the manuscript. All authors read and approved the version.

## Funding

This study was supported by Project BIOSAFE funded by FEDER through the Programa Operacional Factores de Competitividade–COMPETE, by the Fundação para a Ciência e a Tecnologia (FCT, Portugal) - Grant LISBOA-01-0145-FEDER-030713, PTDC/CAL-EST/30713/2017 and by FCT through grants UI/BD/151061/2021 (CM) and 2021.05063.BD (CF) and funds to GHTM (UID/04413/2020) and the CIISA Project (UID/CVT/00276/2020).

## Conflict of interest

The authors declare that the research was conducted in the absence of any commercial or financial relationships that could be construed as a potential conflict of interest.

## Publisher’s note

All claims expressed in this article are solely those of the authors and do not necessarily represent those of their affiliated organizations, or those of the publisher, the editors and the reviewers. Any product that may be evaluated in this article, or claim that may be made by its manufacturer, is not guaranteed or endorsed by the publisher.

## Footnotes

^1^ http://www.bioscand.se/nri/

^2^ https://www.megasoftware.net

^3^ https://pubmlst.org/bigsdb?db=pubmlst_spseudintermedius_seqdef

^4^ https://online.phyloviz.net/index
