# Supplementary material for: Genetic diversity and antimicrobial resistance profiles of Staphylococcus pseudintermedius associated with skin and soft-tissue infections in companion animals in Lisbon, Portugal
Source: Front Microbiol. 2023 Apr 17;14:1167834. doi: 10.3389/fmicb.2023.1167834 (PMC10149759; doi:10.3389/fmicb.2023.1167834)
Supplement: Supplementary file 1 [file Data_Sheet_1.docx]

Supplementary Material

**Genetic diversity and antimicrobial resistance profiles of *Staphylococcus pseudintermedius* associated with skin and soft-tissue infections in companion animals in Lisbon, Portugal.**

**Catarina Morais^1^**, **Sofia Santos Costa^1^**, **Marta Leal^1^, Bárbara Ramos^1^, Mariana Andrade^1^, Carolina Ferreira^1^, Patrícia Abrantes^1^, Constança Pomba^2,3^, Isabel Couto^1^**^*^

*** Correspondence:** Isabel Couto; [icouto@ihmt.unl.pt](mailto:icouto@ihmt.unl.pt)

**Supplementary Table 1**. **Sampling origin and host of the *Staphylococcus pseudintermedius* associated with SSTIs in companion animals analyzed in this study (n=155).**

| **Isolate** | **Biological sample** | **Host** | **Year** |
| --- | --- | --- | --- |
| Laboratory 1 | | | |
| BIOS-V7 | Skin swab | Dog | 2015 |
| BIOS-V10 | Skin swab | Dog | 2018 |
| BIOS-V11^B^ | Skin swab | Dog | 2018 |
| BIOS-V12^D^ | Skin swab | Dog | 2018 |
| BIOS-V13 | Paw swab | Dog | 2018 |
| BIOS-V14 | Skin swab | Dog | 2018 |
| BIOS-V15 | Skin swab | Dog | 2018 |
| BIOS-V16 | Interdigital swab | Dog | 2018 |
| BIOS-V18 | Skin swab | Dog | 2018 |
| BIOS-V25 | Skin swab | Dog | 2016 |
| BIOS-V26 | Biopsy | Dog | 2017 |
| BIOS-V27 | Skin swab | Dog | 2018 |
| BIOS-V28 | Skin swab | Dog | 2018 |
| BIOS-V29 | Interdigital swab | Dog | 2018 |
| BIOS-V32 | Skin swab | Dog | 2018 |
| BIOS-V34^B^ | Skin swab | Dog | 2018 |
| BIOS-V36 | Skin biopsy | Dog | 2017 |
| BIOS-V37^E^ | Skin swab | Dog | 2018 |
| BIOS-V38 | Skin swab | Dog | 2018 |
| BIOS-V39 | Skin biopsy | Dog | 2016 |
| BIOS-V40 | Skin swab | Dog | 2016 |
| BIOS-V48 | Secretion | Dog | 2014 |
| BIOS-V49 | Skin swab | Dog | 2015 |
| BIOS-V50^F^ | Pustule | Dog | 2015 |
| BIOS-V52^E^ | Skin biopsy | Dog | 2017 |
| BIOS-V53^A^ | Skin swab | Dog | 2015 |
| BIOS-V54 | Skin swab | Dog | 2018 |
| BIOS-V55 | Skin swab | Dog | 2014 |
| BIOS-V56 | Skin swab | Dog | 2014 |
| BIOS-V57 | Skin swab | Dog | 2015 |
| BIOS-V58^G^ | Skin swab | Dog | 2014 |
| BIOS-V59 | Skin swab | Dog | 2017 |
| BIOS-V64 | Interdigital swab | Dog | 2018 |
| BIOS-V65 | Skin swab | Dog | 2018 |

(A-H): Isolates collected from the same animal.

**Supplementary Table 1 (continued).**

| **Isolate** | **Biological sample** | **Host** | **Year** |
| --- | --- | --- | --- |
| BIOS-V66 | Lip crease swab | Dog | 2015 |
| BIOS-V67 | Skin swab | Dog | 2018 |
| BIOS-V68 | Armpit skin swab | Dog | 2015 |
| BIOS-V71 | Pustule swab | Dog | 2015 |
| BIOS-V72 | Skin swab | Dog | 2018 |
| BIOS-V73 | Skin swab | Dog | 2017 |
| BIOS-V77 | Swab | Dog | 2015 |
| BIOS-V78 | Scrotal swab | Dog | 2015 |
| BIOS-V79 | Skin swab | Dog | 2015 |
| BIOS-V82^F^ | Epidermal scab swab | Dog | 2015 |
| BIOS-V83 | Abdominal area pustule swab | Dog | 2015 |
| BIOS-V84 | Pustule swab | Dog | 2015 |
| BIOS-V87 | Swab | Dog | 2014 |
| BIOS-V88^G^ | Scab swab | Dog | 2015 |
| BIOS-V89^H^ | Skin- granuloma | Dog | 2015 |
| BIOS-V90 | Skin swab | Dog | 2015 |
| BIOS-V92 | Skin swab | Dog | 2005 |
| BIOS-V96^C^ | Swab | Dog | 2015 |
| BIOS-V97 | Suture swab | Dog | 2015 |
| BIOS-V99^H^ | Chronic wound swab | Dog | 2015 |
| BIOS-V101 | Skin swab | Dog | 2017 |
| BIOS-V102 | Skin swab | Dog | 2015 |
| BIOS-V103 | Interdigital swab | Dog | 2015 |
| BIOS-V104 | Fistula swab | Dog | 2014 |
| BIOS-V105^A^ | Skin swab | Dog | 2017 |
| BIOS-V106 | Deep biopsy | Dog | 2017 |
| BIOS-V108 | Skin granuloma | Dog | 2015 |
| BIOS-V110 | Skin swab | Dog | 2015 |
| BIOS-V113 | Abdominal area pustule | Dog | 2018 |
| BIOS-V114 | Dermatitis fistula in hind limb | Dog | 2015 |
| BIOS-V116^C^ | Skin pustules | Dog | 2015 |
| BIOS-V117 | Skin swab | Dog | 2018 |
| BIOS-V119 | Skin swab | Dog | 2018 |
| BIOS-V120 | Skin swab | Dog | 2017 |
| BIOS-V121 | Skin swab | Dog | 2018 |
| BIOS-V122 | Paw swab | Dog | 2018 |
| BIOS-V123 | Skin exudate | Dog | 2015 |
| BIOS-V124 | Perilabial swab | Dog | 2015 |
| BIOS-V125 | Skin swab | Dog | 2015 |
| BIOS-V127^C^ | Skin swab | Dog | 2016 |
| BIOS-V130 | Paracostal abscess | Rabbit | 2003 |
| BIOS-V131 | Finger swab | Dog | 2014 |
| BIOS-V132 | Chronic wound swab | Dog | 2015 |
| BIOS-V133 | Skin swab | Dog | 2014 |
| BIOS-V134 | Interdigital swab | Dog | 2014 |
| BIOS-V135 | Pustule swab | Dog | 2015 |
| BIOS-V136 | Perilabial swab | Dog | 2015 |
| BIOS-V137 | Skin swab | Dog | 2014 |
| BIOS-V138 | Paw swab | Dog | 2018 |
| BIOS-V140 | Skin swab | Dog | 2015 |
| BIOS-V141 | Skin swab | Dog | 2015 |

(A-H): Isolates collected from the same animal.

**Supplementary Table 1 (continued).**

| **Isolate** | **Biological sample** | **Host** | **Year** |
| --- | --- | --- | --- |
| BIOS-V142 | Skin swab after disinfection | Dog | 2014 |
| BIOS-V143^D^ | Hind limb lesion swab | Dog | 2014 |
| BIOS-V144^D^ | Pustule swab | Dog | 2014 |
| BIOS-V145 | Ventral pustule | Dog | 2018 |
| BIOS-V146 | Skin swab | Dog | 2015 |
| Laboratory 2 |  |  |  |
| BIOS-V162 | Nodular lesion swab | Dog | 2018 |
| BIOS-V164 | Surgical wound swab | Dog | 2018 |
| BIOS-V167 | Submandibular abscess swab | Dog | 2018 |
| BIOS-V170 | Arm abscess swab | Dog | 2018 |
| BIOS-V175 | Skin swab | Dog | 2018 |
| BIOS-V176 | Skin swab | Dog | 2018 |
| BIOS-V179 | Skin swab | Dog | 2018 |
| BIOS-V182 | Subcutaneous swab | Dog | 2018 |
| BIOS-V188 | Hind limb abscess swab | Dog | 2018 |
| BIOS-V189 | Pyoderma swab | Dog | 2018 |
| BIOS-V190 | Papule in armpit | Dog | 2018 |
| BIOS-V194 | Skin swab | Dog | 2018 |
| BIOS-V195 | Skin swab | Dog | 2018 |
| BIOS-V196 | Skin swab | Dog | 2018 |
| BIOS-V207 | Thigh wound swab | Cat | 2018 |
| BIOS-V211 | Skin swab | Dog | 2018 |
| BIOS-V212 | Interdigital swab | Dog | 2018 |
| BIOS-V213 | Amputation thigh material swab | Dog | 2018 |
| BIOS-V214 | Skin swab | Dog | 2018 |
| BIOS-V217 | Skin swab | Dog | 2018 |
| BIOS-V218 | Skin swab | Dog | 2018 |
| BIOS-V219 | Skin swab- right armpit | Dog | 2018 |
| BIOS-V221 | Skin swab | Dog | 2018 |
| BIOS-V223 | Surgical site swab | Dog | 2018 |
| BIOS-V224 | Face mass swab | Dog | 2018 |
| BIOS-V225 | Interdigital swab | Dog | 2018 |
| BIOS-V226 | Pelvic limb swab | Dog | 2018 |
| BIOS-V227 | Pustule swab | Dog | 2018 |
| BIOS-V228 | Skin swab | Dog | 2018 |
| BIOS-V230 | Pelvic limb biopsy | Dog | 2018 |
| BIOS-V231 | Skin swab | Dog | 2018 |
| BIOS-V233 | Papule swab | Dog | 2018 |
| BIOS-V234 | Skin swab | Dog | 2018 |
| BIOS-V235 | Skin pustule swab | Dog | 2018 |
| BIOS-V236 | Skin swab | Dog | 2018 |
| BIOS-V237 | Torso swab | Dog | 2018 |
| BIOS-V238 | Armpit skin swab | Dog | 2018 |
| BIOS-V239 | Limb swab | Dog | 2018 |
| BIOS-V240 | Fracture wound swab | Cat | 2018 |
| BIOS-V241 | Granuloma swab | Dog | 2018 |
| BIOS-V242 | Biopsy swab | Dog | 2018 |
| BIOS-V244 | Skin swab | Dog | 2018 |
| BIOS-V247 | Callus swab | Dog | 2018 |
| BIOS-V249 | Skin swab | Dog | 2018 |
| BIOS-V251 | Exudate swab | Dog | 2018 |

(A-H): Isolates collected from the same animal.

**Supplementary table 1 (continued).**

| **Isolate** | **Biological sample** | **Host** | **Year** |
| --- | --- | --- | --- |
| BIOS-V259 | Skin swab | Dog | 2018 |
| BIOS-V260 | Foreskin swab | Dog | 2018 |
| BIOS-V262 | Dermal punch | Dog | 2018 |
| BIOS-V263 | Papule | Dog | 2018 |
| BIOS-V264 | Interdigital swab | Dog | 2018 |
| BIOS-V268 | Skin exudate swab | Dog | 2018 |
| BIOS-V270 | Skin wound swab | Dog | 2018 |
| BIOS-V273 | Wound swab | Dog | 2018 |
| BIOS-V276 | Thigh swab | Dog | 2018 |
| BIOS-V280 | Surgical material | Dog | 2018 |
| BIOS-V281 | Skin swab | Dog | 2018 |
| BIOS-V285 | Skin swab | Dog | 2018 |
| BIOS-V286 | Pustule swab | Dog | 2018 |
| BIOS-V287 | Skin swab | Dog | 2018 |
| BIOS-V290 | Chin swab | Dog | 2018 |
| BIOS-V292 | Suture swab | Cat | 2018 |
| BIOS-V297 | Skin swab | Dog | 2018 |
| BIOS-V298 | Skin swab | Dog | 2018 |
| BIOS-V299 | Interdigital furunculosis swab | Dog | 2018 |
| BIOS-V302 | Skin swab | Dog | 2018 |

(A-H): Isolates collected from the same animal.

**Supplementary Table 2**. **Primers used in the study.**

| **Target gene** | **Primers** | **Nucleotide Sequence (5’-3’)** | **Amplicon Size (bp)** | **Reference** |
| --- | --- | --- | --- | --- |
| ***S. pseudintermedius* identification** | | | | |
| *spsJ* | stapse_Fw | ACCAAG GCCTGTAAGTAAAGCACC | 198 | Verstappen *et al.*, 2017 |
|  | stapse_Rv | TCTCTTTCAACATCGGCATCAACGC |  |  |
| **Screening of resistance genes and mutations** | | | | |
| *blaZ* | blaZ_Fw | GATAAGAGATTTGCCTATGC | 533 | Milheiriço *et al*., 2011 |
|  | blaZ_Rv | GCATATGTTATTGCTTGACC |  |  |
| *mecA* | mecA_Fw | GGTCCCATTAACTCTGAAG | 1040 | Petinaki *et al*., 2001 |
|  | mecA_Rv | AGTTCTGCAGTACCGGATTTGC |  |  |
| *erm*(A) | erm(A)_Fw | AAGCGGTAAACCCCTCTGAG | 442 | Jensen *et al*., 2002 |
|  | erm(A)_Rv | TCAAAGCCTGTCGGAATTGG |  |  |
| *erm*(B) | erm(B)_Fw | TGGAACAGGTAAAGGGCATT | 433 | Costa *et al*., 2016 |
|  | erm(B)_Rv | TGTGGTATGGCGGGTAAGTT |  |  |
| *erm*(C) | erm(C)_Fw | TCGTAACTGCCATTGAAATA | 348 | Costa *et al*., 2016 |
|  | erm(C)_Rv | TCACTTTAGGTTTAGGATGAAA |  |  |
| *vga*(C) | vga(C)_Fw | ACGAATAAAGGGATCGAAGC | 510 | Ferreira *et al*., 2021 |
|  | vga(C)_Rv | AGCACATGCACAGGTTTGTA |  |  |
| *aadD* | aadD_Fw | GGAAGCAGAGTTCAGCCATG | 266 | Kobayashi *et al*., 2001 |
|  | aadD_Rv | TGCCTGCATATTCAAACAGC |  |  |
| *aph3-IIIa* | aph3-IIIa_Fw | CCGCTGCGTAAAAGATAC | 609 | Perreten *et al*., 2005 |
|  | aph3-IIIa_Rv | GTCATACCACTTGTCCGC |  |  |
| *aacA-aphD* | aacA-aphD_Fw | CAGAGCCTTGGGAAGATGAAG | 348 | Vakulenko *et al*., 2003 |
|  | aacA-aphD_Rv | CCTCGTGTAATTCATGTTCTGGC |  |  |
| *cat*_pC221_ | catpC221_Fw | ATTTATGCAATTATGGAAGTTG | 435 | Schnellmann *et al*., 2006 |
|  | catpC221_Rv | TGAAGCATGGTAACCATCAC |  |  |
| *dfrA*(S1) | dfrA_Fw | CACTTGTAATGGCACGGAAA | 270 | Argudin *et al*., 2011 |
|  | dfrA_Rv | CGAATGTGTATGGTGGAAAG |  |  |
| *dfr*(G) | dfr(G)_Fw | TTTCTTTGATTGCTGCGATG | 501 | Ruzauskas *et al*., 2015 |
|  | dfr(G)_Rv | AACGCACCCGTTAACTCAAT |  |  |
| *fusB* | fusB_Fw | TCATATAGATGACGATATTG | 496 | Castanheira *et al*., 2010 |
|  | fusB_Rv | ACAATGAATGCTATCTCGAC |  |  |
| *fusC* | fusC_Fw | GATATTGATATCTCGGACTT | 128 | Castanheira *et al*., 2010 |
|  | fusC_Rv | AGTTGACTTGATGAAGGTAT |  |  |
| *tet(*K) | tet(K)_Fw | GTAGCGACAATAGGTAATAGT | 360 | Strommenger *et al.*, 2003 |
|  | tet(K)_Rv | GTAGTGACAATAAACCTCCTA |  |  |

**Supplementary Table 2 (continued)**.

| **Target gene** | **Primers** | **Nucleotide Sequence (5’-3’)** | **Amplicon Size (bp)** | **Reference** |
| --- | --- | --- | --- | --- |
| *tet(*M) | tet(M)_Fw | GTTAAATAGTGTTCTTGGAG | 657 | Aarestrup *et al*., 2000 |
|  | tet(M)_Rv | CTAAGATATGGCTCTAACAA |  |  |
| *tet*(L) | tet(L)_Fw | GTCGGTAATTGGGTTTGTTG | 421 | Costa *et al*., 2021 |
|  | tet(L)_Rv | TGACAGCACGCTAACGATAA |  |  |
| *grl*A | grlA_Fw | CAAGAGCGTGCTTTRCCT | 300 | Costa *et al*., 2021 |
|  | grlA_Rv | CTGACTYAATTTCGCTTCAG |  |  |
| *gyrA* | gyrA_Fw | ATGAGTGTTATYGTRTCTCGT | 261 | Costa *et al*., 2021 |
|  | gyrA_Rv | CATMGAACCRAAGTTACCTTG |  |  |
| **MLST** | | | | |
| *ack* | ack_Fw | CACCACTTCACAACCCAGCAAACT | 680 | Solyman *et al*., 2013 |
|  | ack_Rv | AACCTTCTAATACACGCGCACGCA |  |  |
| *cpn60* | cpn60_Fw | GCGACTGTACTTGCACAAGCA | 552 | Bannoehr *et al*., 2007 |
|  | cpn60_Rv | AACTGCAACCGCTGTAAATG |  |  |
| *fdh* | fdh_Fw | TGCGATAACAGGATGTGCTT | 408 | Solyman *et al*., 2013 |
|  | fdh_Rv | CTTCTCATGATTCACCGGC |  |  |
| *pta* | pta_Fw | GTGCGTATCGTATTACCAGAAGG | 570 | Bannoehr *et al*., 2007 |
|  | pta_Rv | GCAGAACCTTTTGTTGAGAAGC |  |  |
| *purA* | purA_Fw | GATTACTTCCAAGGTATGTTT | 490 | Solyman *et al*., 2013 |
|  | purA_Rv | TCGATAGAGTTAATAGATAAGTC |  |  |
| *sar* | sar_Fw | GGATTTAGTCCAGTTCAAAATTT | 521 | Solyman *et al*., 2013 |
|  | sar_Rv | GAACCATTCGCCCCATGAA |  |  |
| *tuf* | tuf_Fw | CAATGCCACAAACTCG | 500 | Bannoehr *et al*., 2007 |
|  | tuf_Rv | GCTTCAGCGTAGTCTA |  |  |


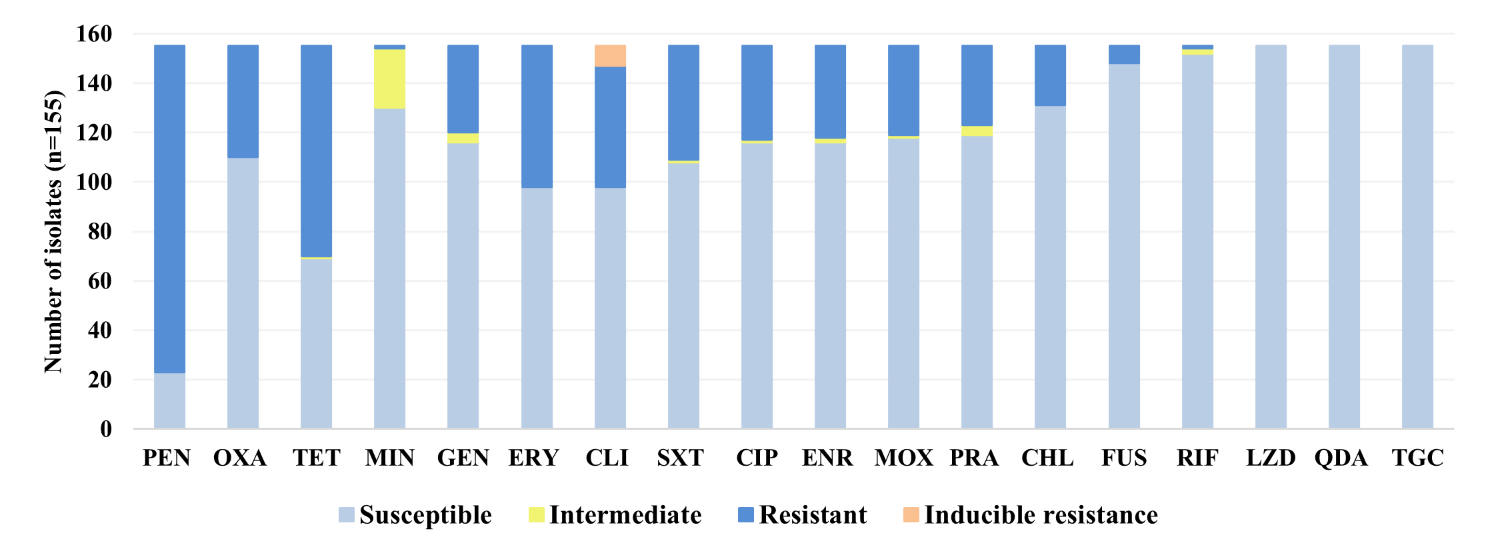


**Supplementary Figure 1. Distribution of antimicrobial susceptibility phenotypes among the *S. pseudintermedius* collection (n=155).**

PEN: penicillin; OXA: oxacillin; TET: tetracycline; MIN: minocycline; GEN: gentamycin; ERY: erythromycin; CLI: clindamycin; SXT: trimethoprim-sulfamethoxazole; CIP: ciprofloxacin; ENR: enrofloxacin; MOX: moxifloxacin; PRA: pradofloxacin; CHL: chloramphenicol; FUS: fusidic acid; RIF: rifampicin; LZD: linezolid; QDA: quinupristin-dalfopristin; TGC: tigecycline.

**CO_WT_**

**WT**

**NWT**

**Supplementary Figure 2. Distributions of zones of inhibition of antimicrobials without available breakpoints for the 155 *S. pseudintermedius* isolates and respective cut-off values determined according to the Normalized Resistance Interpretation method.** Dark columns represent the distribution of the zones of inhibition, and the brown line indicates the NRI generated normalized distribution of the wild-type populations. The CO_WT_ values are indicated through a dashed vertical line and the wild-type and non-wild type populations are indicated by arrows. CO_WT_: cut-off value; WT: wild-type; NWT: non-wildtype.

**WT**

**NWT**


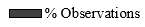

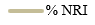


**WT**

**NWT**

**CO_WT_**

**WT**

**NWT**

**CO_WT_**

**CO_WT_**

**Supplementary Figure 2 (continued). Distributions of zones of inhibition of antimicrobials without available breakpoints for the 155 *S. pseudintermedius* isolates and respective cut-off values determined according to the Normalized Resistance Interpretation method.** Dark columns represent the distribution of the zones of inhibition, and the brown line indicates the NRI generated normalized distribution of the wild-type populations. The CO_WT_ values are indicated through a dashed vertical line and the wild-type and non-wildtype populations are indicated by arrows. CO_WT_: cut-off value; WT: wild-type; NWT: non-wild type.


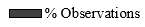

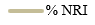


**WT**

**NWT**

**WT**

**NWT**

**WT**

**NWT**

**WT**

**NWT**

**CO_WT_**

**CO_WT_**

**CO_WT_**

**CO_WT_**

**WT**

**NWT**

**CO_WT_**


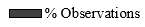

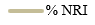


**Supplementary Figure 2 (continued). Distributions of zones of inhibition of antimicrobials without available breakpoints for the 155 *S. pseudintermedius* isolates and respective cut-off values determined according to the Normalized Resistance Interpretation method.** Dark columns represent the distribution of the zones of inhibition, and the brown line indicates the NRI generated normalized distribution of the wild-type populations. The CO_WT_ values are indicated through a dashed vertical line and the wild-type and nonwild-type populations are indicated by arrows. CO_WT_: cut-off value; WT: wild-type; NWT: non-wild type.


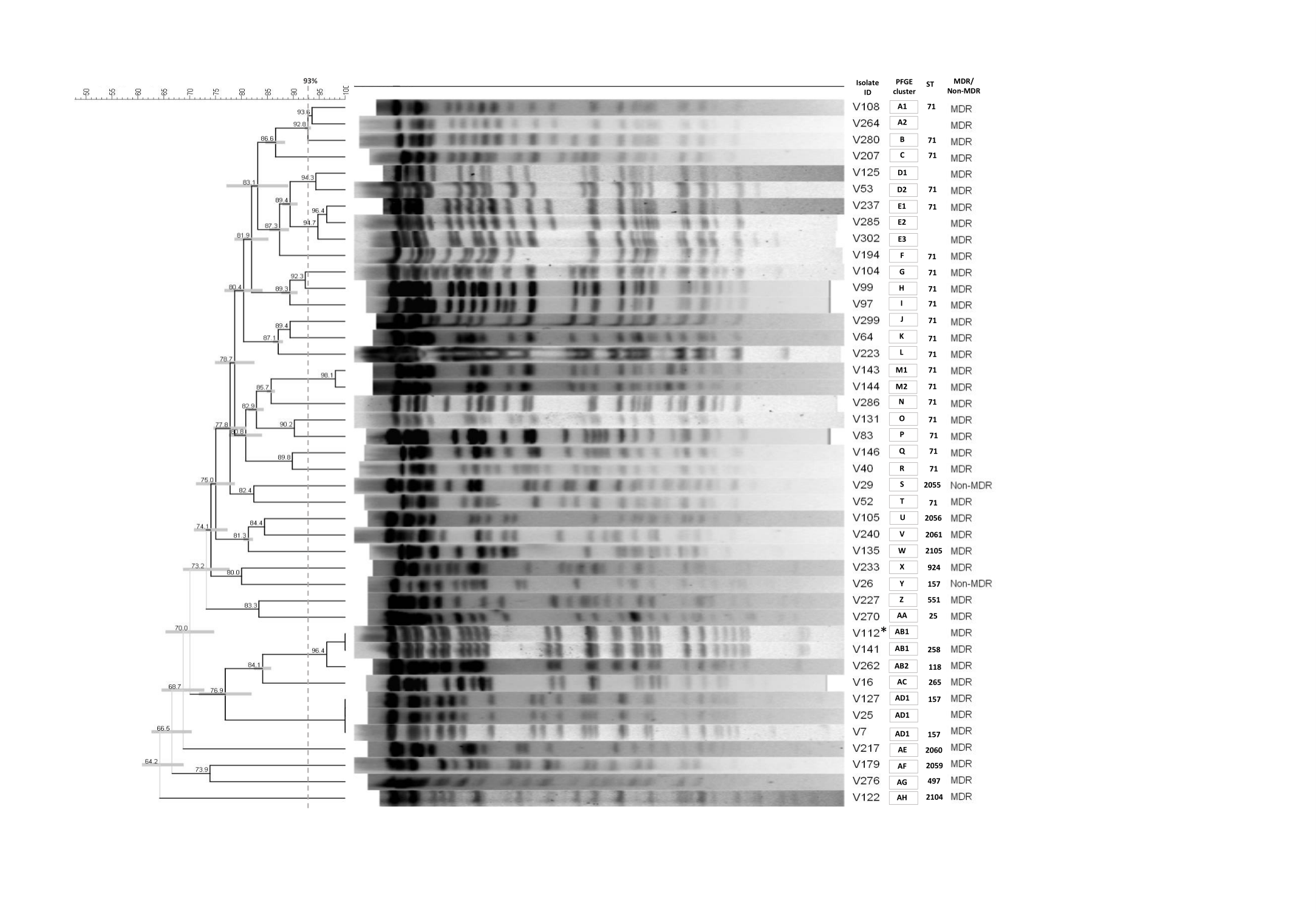


**Supplementary Figure 3.** ***Sma*I macrorestriction profiles of MRSP resolved by PFGE.**

Details of cluster analysis are provided in Figure 3 of the main text. Each type is designed by capital letter(s), with numbers indicating subtypes. * Isolates V112 and V141 were collected from the same animal. Because they were pheno- and genotypically indistinguishable; isolate V112 was not included in the final analysis.

# References

Aarestrup F.M., Agerso Y., Gerner-Smidt P., Madsen M., Jensen L.B. (2000). Comparison of antimicrobial resistance phenotypes and resistance genes in *Enterococcus faecalis* and *Enterococcus faecium* from humans in the community, broilers, and pigs in Denmark. Diagn. Microbiol. Infect. Dis. 37:127–137.

Argudín M.A., Tenhagen B.A., Fetsch A., Sachsenröder J., Käsbohrer A., Schroeter A., *et al*. (2011). Virulence and resistance determinants of German *Staphylococcus aureus* ST398 isolates from nonhuman sources. Appl. Environ. Microbiol. 77(9):3052-3060. doi: 10.1128/AEM.02260-10

Bannoehr J., Zakour N.L.B., Walker A.S., Guardabassi L., Thoday K.L., van den Broek A.H.M., *et al*. (2007). Population genetic structure of the *Staphylococcus intermedius* Group: Insights into *agr* diversification and the emergence of methicillin-resistant strains. J. Bacteriol. 189(23):8685-8692. doi:10.1128/JB.01150-07

Castanheira M., Watters A.A., Bell J.M., Turnidge J.D., Jones R.N. (2010). Fusidic acid resistance rates and prevalence of resistance mechanisms among *Staphylococcus* spp. isolated in North America and Australia, 2007-2008. Antimicrob. Agents Chemother. 54(9):3614-3617. doi: 10.1128/AAC.01390-09

Costa S.S., Palma C., Kadlec K., Fessler A.T., Viveiros M., Melo-Cristino J., *et al*. (2016). Plasmid-borne antimicrobial resistance of *Staphylococcus aureus* isolated in a hospital in Lisbon, Portugal. Microb. Drug Resist. 22(8):617-626. doi: 10.1089/mdr.2015.0352

Costa S.S., Oliveira V., Serrano M., Pomba C., Couto I. (2021). Phenotypic and molecular traits of *Staphylococcus coagulans* associated with canine skin infections in Portugal. Antibiotics. 10:518. doi: 10.1089/mdr.2020.0402

Ferreira C., Costa S.S., Serrano M., Oliveira K., Trigueiro G., Pomba C., *et al*. (2021). Clonal lineages, antimicrobial resistance, and PVL carriage of *Staphylococcus aureus* associated to skin and soft-tissue infections from ambulatory patients in Portugal. Antibiotics. 10:345. doi: 10.3390/antibiotics10040345

Kobayashi N., Alam M., Nishimoto Y., Urasawa S., Uehara N., Watanabe N. (2001). Distribution of aminoglycoside resistance genes in recent clinical isolates of *Enterococcus faecalis*, *Enterococcus faecium* and *Enterococcus avium*. Epidemiol. Infect. 126:197-204.

Jensen L.B., Hammerum A.M., Bager F., Aarestrup F.M. (2002). Streptogramin resistance among *Enterococcus faecium* isolated from production animals in Denmark in 1997. Microb. Drug Resist. 8(4):369-374. doi: 10.1089/10766290260469642

Milheiriço C., Portelinha A., Krippahl L., Lencastre H., Oliveira D.C. (2011). Evidence for a purifying selection acting on the ß-lactamase locus in epidemic clones of methicillin-resistant *Staphylococcus aureus*. BMC Microbiol. 11:76. doi:10.1186/1471-2180-11-76

Perreten V., Vorlet-Fawer L., Slickers P., Ehricht R., Kuhnert P., Frey J. (2005). Microarray-based detection of 90 antibiotic resistance genes of Gram-positive bacteria. J. Clin. Microbiol. 43(5):2291-2302. doi: 10.1128/JCM.43.5.2291–2302.2005

Petinaki E., Arvaniti A., Dimitracopoulos G., Spiliopoulou I. (2001). Detection of *mecA*, *mecR1* and *mecI* genes among clinical isolates of methicillin-resistant staphylococci by combined polymerase chain reaction. J. Antimicrob. Chemother. 2001; 47:297-304.

Ruzauskas M., Couto N., Pavilonis A., Klimiene I., Siugzdiniene R., Virgailis M. (2016). Characterization of *Staphylococcus pseudintermedius* isolated from diseased dogs in Lithuania. Pol. J. Vet. Sci. 19(1):7-14. doi: 10.1515/pjvs-2016-0002

Schnellmann C., Gerber V., Rossano A., Jaquier V., Panchaud Y., Doherr M.G., *et al*. (2006). Presence of new *mecA* and *mph*(C) variants conferring antibiotic resistance in *Staphylococcus* spp. isolated from the skin of horses before and after clinic admission. J. Clin. Microbiol. 44(12):4444-4454. doi: 10.1128/JCM.00868-06

Strommenger B., Kettlitz C., Werner G., Witte W. (2003). Multiplex PCR assay for simultaneous detection of nine clinically relevant antibiotic resistance genes in *Staphylococcus aureus*. J. Clin. Microbiol. 41(9):4089-4094. doi: 10.1128/JCM.41.9.4089–4094.2003

Solyman S.M., Black C.C., Duim B., Perreten V., van Duijkeren E., Wagenaar J.A., *et al.* (2013). Multilocus sequence typing for characterization of *Staphylococcus pseudintermedius*. J. Clin. Microbiol. 51:306-310. doi: 10.1128/JCM.02421-12

Vakulenki S.B., Donabedian S.M., Voskresenskiy A.M., Zervos M.J., Lerner S.A., Chow J.W. (2003). Multiplex PCR for detection of aminoglycoside resistance genes in Enterococci. Antimicrob. Agents Chemother. 47(4):1423-1426. doi: 10.1128/AAC.47.4.1423–1426.2003

Verstappen K.M., Huijbregts L., Spaninks M., Wagenaar J.A., Fluit A.C., Duim B. (2017). Development of a real-time PCR for detection of *Staphylococcus pseudintermedius* using a novel automated comparison of whole-genome sequences. PLOS one. 12(8):e0183925. doi: 10.1371/journal.pone.0183925
